# COVID-19 outbreak in a military unit in Korea

**DOI:** 10.4178/epih.e2021065

**Published:** 2021-09-08

**Authors:** Chanhee Kim, Young-Man Kim, Namwoo Heo, Eunjung Park, Sojin Choi, Sehyuk Jang, Nayoung Kim, Donghyok Kwon, Young-Joon Park, Byeongseop Choi, Beomman Ha, Kyounghwa Jung, Changbo Park, Sejin Park, Heeyoung Lee

**Affiliations:** 1 Infectious Disease Control Center, Gyeonggi Provincial Government, Suwon, Korea; 2 Central Disease Control Headquarters, Korea Disease Control and Prevention Agency, Cheongju, Korea; 3 Regional Center for Disease Control and Prevention, Korea Disease Control and Prevention Agency, Seoul, Korea; 4 Republic of Korea Armed Forces Medical Command, Korea Army, Seongnam, Korea; 5 Republic of Korea Army Headquarters, Korea Army, Gyerong, Korea; 6 Republic of Korea Armed Forces Epidemiologic Investigation Center, Korea Army, Seongnam, Korea; 7 Center for Preventive Medicine and Public Health, Seoul National University Bundang Hospital, Seongnam, Korea

**Keywords:** COVID-19, SARS-CoV-2, Disease outbreaks, Military facilities

## Abstract

**OBJECTIVES:**

This study presents the response of a military unit to an outbreak of coronavirus disease 2019 (COVID-19) in Gyeonggi Province. As soon as 2 soldiers were identified as index cases, the infectious disease investigators of the Gyeonggi Provincial Government, Korea Disease Control and Prevention Agency, and the Armed Forces Epidemiologic Investigation Center discussed the investigation and response plan for an imminent massive outbreak.

**METHODS:**

The joint immediate response team (IRT) conducted interviews with confirmed COVID-19 patients, reviewed their medical records, performed contact tracing using global positioning system data, and undertook a field investigation. For risk assessment, the joint IRT visited all 8 sites of the military units and the army chaplain’s church to evaluate the transmission risk at each site. The evaluation items included the size of the site, the use of air conditioning, whether windows were opened, and whether masks were worn. Pooled testing was used for the low-risk population to quickly detect the spread of COVID-19 in the military base.

**RESULTS:**

One day before the symptom onset of the index case, the lecturer and >50% of the attendees were infected with COVID-19 while attending a lecture that lasted 2 hours and 30 minutes. Attendees were not wearing masks and were in a poorly ventilated room.

**CONCLUSIONS:**

Since COVID-19 can be spread before symptom onset, contact tracing must be performed to investigate potential exposures prior to symptom onset and to manage any exposed persons.

## INTRODUCTION

Since the first identified case of coronavirus disease 2019 (COVID-19) in Korea on January 20, 2020, the number of cases in Korea has sharply increased [1-3]. In addition to individually transmitted cases, cluster cases at locations including call centers, dance classes, and nightclubs have also substantially contributed to the disease burden of COVID-19 in Korea [4-6].

The military population is well-known for its vulnerability to various types of communicable diseases due to its collective living environment with close quarters [7-9]. In Korea, outbreaks of tuberculosis and respiratory syncytial virus have been reported among the military population, demonstrating its vulnerability to communicable diseases [7,8,10].

However, research regarding the COVID-19 outbreak in military settings has rarely been reported. Therefore, this study aimed to introduce lessons learned and share experiences from responding to a COVID-19 outbreak in a military unit in Gyeonggi Province, Korea. Furthermore, since an outbreak of COVID-19 involving a military unit has never before been reported in Korea, the results of our study may help to establish policies to control and manage collective environments, such as military units.

On July 20, 2020, 2 soldiers in an army combat service battalion in Pocheon City, Gyeonggi Province were suspected of having COVID-19. Both were tested and received positive COVID-19 results the next day. The infectious disease investigators of the Gyeonggi Provincial Government, Korea Disease Control and Prevention Agency (KDCA) and the Armed Forces Epidemiologic Investigation Center discussed the investigation plan and commenced broad and quick screening to identify further cases of COVID-19 due to the possibility of an imminent massive outbreak.

## MATERIALS AND METHODS

### Outbreak recognition

The relevant combat service support battalion in Pocheon City, Gyeonggi Province consisted of 250 soldiers. On July 16, 2020, a lecture was held for soldiers scheduled to be discharged, which was attended by 2 instructors and 26 soldiers. After 4 days, on July 2020, 2 soldiers who participated in the lecture reported symptoms of fever and sore throat to the commander. Since these could be COVID-19 symptoms, they were immediately tested for COVID-19 using polymerase chain reaction (PCR) tests and were informed of their positive test results on July 21, 2020. The military authorities then conducted COVID-19 PCR tests on all soldiers within the battalion. This resulted in 12 additional confirmed cases, 11 of whom had also attended the lecture. The exception was a soldier who shared the same barrack with the rest of the confirmed COVID-19 cases. In addition, the 2 visiting instructors were confirmed to be COVID-19-positive on July 22, 2020. The onset of their symptoms was earlier than that of the soldiers. According to previously known modes of transmission of COVID-19, the immediate response team (IRT) hypothesized that the aerosol produced by the lecturers spread the COVID-19 infection to poorly masked soldiers during the class [11,12]. It was then determined that the outbreak could no longer be defined as an internal outbreak, and a joint epidemiologic investigation was launched by the public health and military authorities.

### Case definition and epidemiologic investigations

A confirmed case of COVID-19 was defined based on a positive COVID-19 PCR test result on or after July 21, 2020, for any member or visitor within the 8 sites of the military units. Symptoms or symptom onset were not considered in the case definition.

The joint IRT conducted epidemiologic investigations, which comprised traditional shoe-leather epidemiology (including interviews with confirmed patients and reviews of medical records) and new methods to track contacts using global positioning system (GPS) data and credit card transactions [13]. Closed-circuit televisions (CCTVs) are routinely used in epidemiologic investigations in Korea; however, there were no CCTVs that could be viewed inside the military unit due to security concerns.

### Risk assessment of transmission sites

For risk assessment, the joint IRT visited all 8 sites of the military units and the army chaplain’s church to evaluate the transmission risk at each site. The evaluation items included the size of the site, the use of air conditioning, whether windows were opened, and whether masks were worn by the users. The lecture hall and the church were identified as the primary transmission sites since confirmed cases in the early phase were mostly identified from these locations.

### Allocation of diagnostic resources

Based on the results of the epidemiological investigation and risk assessment by the IRT, a high-risk group of soldiers was recommended to receive individual testing, while pooled testing was performed for low-risk populations. Pooled testing is a method of mixing several samples to form 1 group sample, followed by a reexamination individually to identify the final confirmed case if there is a positive result [14,15]. Group testing can maintain a sensitivity of > 96% even after mixing up to 10 samples [16]. Since this testing protocol can be implemented promptly to detect COVID-19 [17], we used it to quickly find people who were infected with COVID-19 in the military unit in Gyeonggi Province to reduce transmission. As a result, COVID-19 PCR tests for 1,655 soldiers were completed in 72 hours.

### Cooperation between Gyeonggi Province, Korea Disease Control and Prevention Agency, and military authorities

As of July 2020, the outbreak was the first major cluster infection reported in a military unit in Korea. As soon as the outbreak was identified, on July 22, 2020, the day after the first 2 soldiers were confirmed positive, a meeting between Gyeonggi Province, KDCA, and military authorities took place in the health center of Pocheon City. Through discussion, the following main management strategies for the situation were established: interview of the cases, risk assessment of the transmission site, a quick check of GPS and credit card usage history of cases, review of the medical records of cases, and management and PCR testing of the at-risk population.

Gyeonggi Province gathered infectious disease investigators from other cities in Gyeonggi Province to Pocheon City to conduct interviews of the cases. The Gyeonggi Province investigators visited every place in the military unit where the cases had been present to identify the possibility of further transmission. The KDCA immediately registered the information of cases with the Epidemiological Investigation Support System to obtain the GPS and credit card usage information. The KDCA also requested the cooperation of every health center in Korea to perform PCR tests on soldiers who were not in the barrack because they were on vacation or had already been discharged, but were classified as an atrisk population. The health center of Pocheon City traced those soldiers with the results of their PCR tests. Fortunately, none of these soldiers were confirmed cases. With the cooperation of the health authorities, the military authorities provided PCR tests and places for isolation to soldiers.

### Statistical analysis

In addition to the description of the epidemiological investigation process, we analyzed the attack rate, incubation period, and serial interval of all 8 exposed sites. These sites included the career counseling lectures, church, and a barrack (other barracks did not report any additional confirmed cases). Attack rates were calculated by dividing the total number of confirmed cases by the number of persons at each site [18]. Since the military environment is closed and restricted, the exposure periods of each case were definite. Therefore, the incubation period and serial interval were estimated by exact observations. The incubation period was estimated based on the number of days until symptoms started from the day of exposure [19]. The serial interval was the time difference between the day of symptom onset of the suspected infector and that of the infected individual [20]. For field-training, 1 of the 8 investigated sites, it was difficult to define the at-risk population due to limitations of data acquisition; therefore, the attack rate, incubation period, and serial interval were not able to be analyzed.

### Ethics statement

This study was approved by the Institutional Review Board of Seoul National University Bundang Hospital, and it was recognized that the exemption of informed consent was reasonable for the study because it does not contain personal identifiable information (B-2106/688-101).

## RESULTS

In total, 22 confirmed cases were found at the military base: 19 military members, 2 civilian instructors, and 1 civilian in the family of an instructor (Table 1). The mean age of cases was 24.72 years, since most of the soldiers in Korea are drafted and fulfill their military duty during their early 20s. Most of the cases were symptomatic, except 2 cases in soldiers. The commonly reported symptoms of cases included fever, sore throat, dysfunction of smell or taste, myalgia, and chills.

Of the 19 military members confirmed to be COVID-19-positive, 13 (68.4%) contracted the disease at a single lecture located in military Unit C, where 52.0% (13/25) of the lecture participants were confirmed to be positive (Table 2). No cases were reported from other lectures, with the exception of a lecture in Unit D, where 1 confirmed case was identified among 27 attendees, resulting in an attack rate of 3.7%. The average incubation period and serial interval for the total 22 cases were 6.3 days (range, 3-14) and 5.8 days (range, 2-17), respectively.

**Figure 1.**
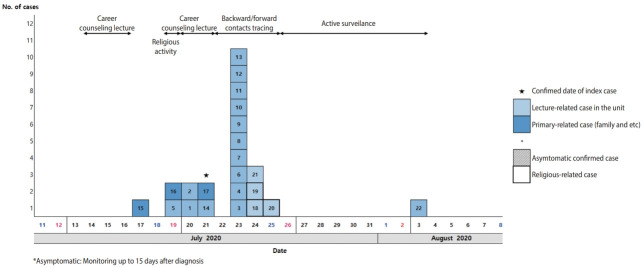
Epidemic curve of confirmed coronavirus disease 2019 (COVID-19) cases in Military units in Pocheon-si, Gyeonggi-do, Korea.

**Figure 2.**
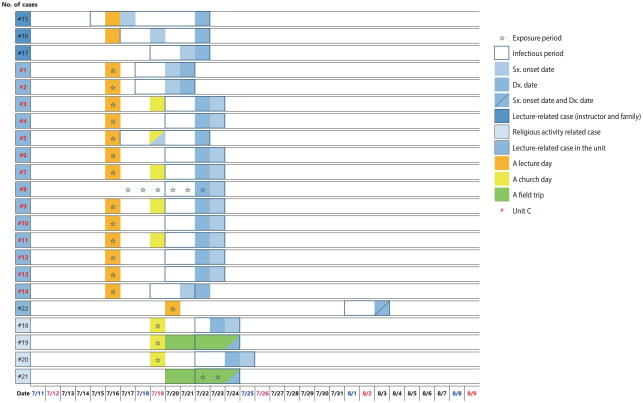
Temporal dynamics of outbreak in Military units in Pocheon-si, Gyeonggi-do, Korea. Sx, symptoms; Dx, diagnosis.

The date of symptom onset for the suspected primary case (one of the instructors) was July 17, 2020. Five lectures were held by the suspected primary case on July 14, 15, 16, 20, and 21, 2020 (Figure 1). Soldiers who attended the July 14, 2020, lecture were excluded from the investigation considering the infectious period of the lecturer. A secondary infection occurred in a soldier who did not attend the lecture on July 16, 2020, but shared the common-use space in a barrack (Figure 2). After the lecture, some of the soldiers who were already infected with the disease, but were not aware of their status, visited the poorly-ventilated and poorly-distanced church, resulting in additional transmission. Finally, the last transmission occurred during field-training, resulting in 1 more confirmed case.

**Figure 3.**
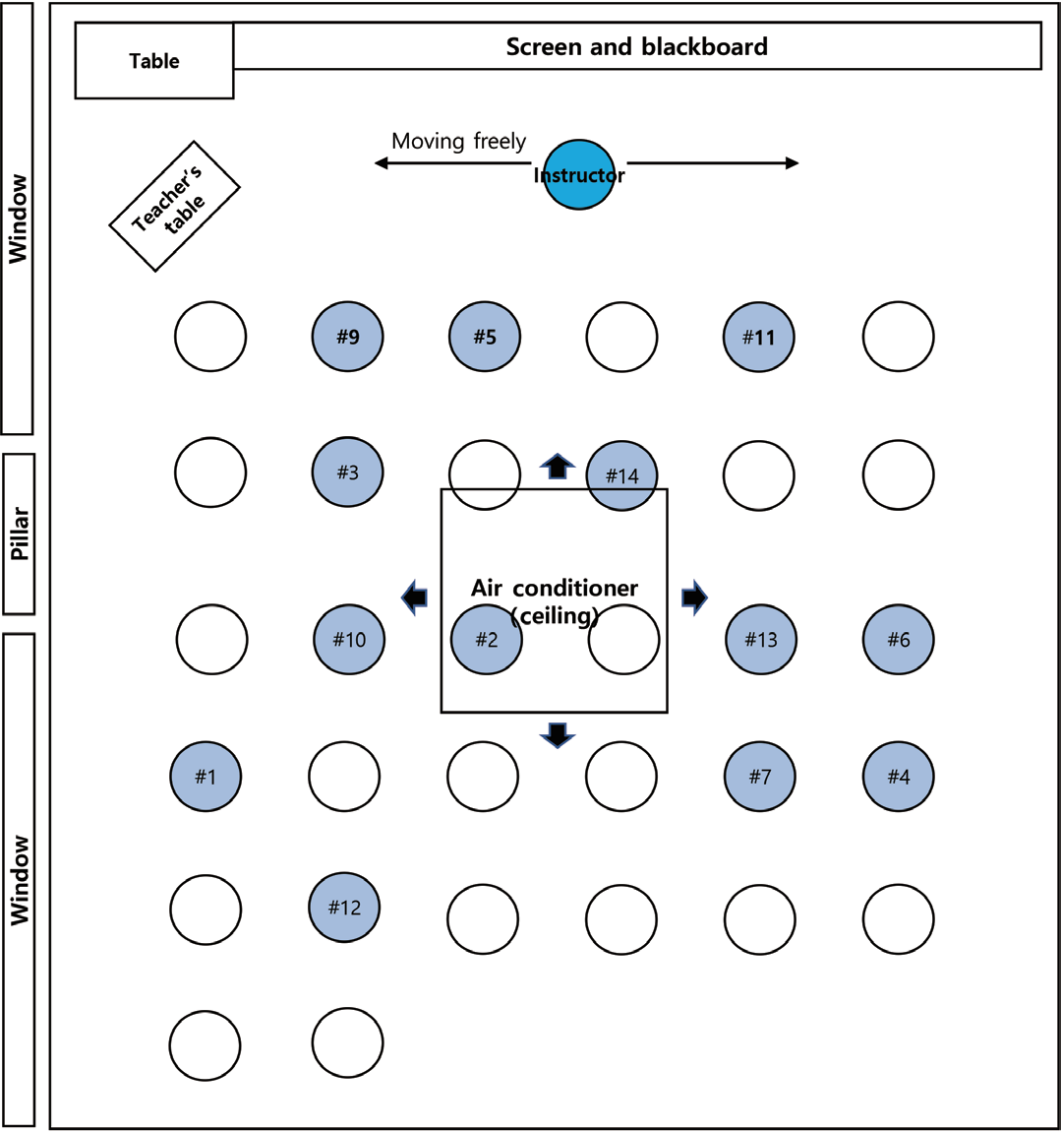
Spread of coronavirus disease 2019 (COVID-19) during a lecture in the classroom of Unit A, Pocheon-si, Gyeonggi-do, Korea.

The area of the classroom in Unit C, where the largest number of confirmed cases was discovered, was 81.6 m^2^, which was smaller than that of the classrooms in other units (Table 2). Moreover, during the lecture, the windows were closed and the air conditioner (ceiling type) was on. As the instructor moved freely inside the lecture room during the lecture, the breeze from the air conditioner was directed from the instructor toward the soldiers, who were not appropriately wearing masks (Figure 3).

The church is another place where substantial transmission took place. Like the lecture room of Unit C, the church was poorly ventilated and not in compliance with the KDCA’s recommendations. Through risk assessment surveillance, the joint IRT found that the chairs in the church were placed too close to each other. During the chapel service, 73 soldiers from three battalions were seated on the ends of each pew to prevent transmission. However, even if the left and right spacing was maintained, the gap between the front and back of the pews was too narrow. In addition, not all soldiers were wearing masks during the chapel service. Transmission at the church resulted in 4 additional confirmed cases, including the field-training case. One COVID-19-confirmed soldier did not attend the chapel service, but participated in the same fieldtraining with another COVID-19-confirmed soldier who visited the church. The joint IRT therefore concluded that he was infected during field-training when all the subunits came together. All soldiers who were potential contacts completed 14 days of quarantine and all received negative COVID-19 PCR test results on August 6, 2020.

The barracks had different conditions compared to the classroom where the lectures took place. Although the barracks are home to soldiers, which might have resulted in additional transmission of the disease (similarly to household transmission), only 1 case was found as a barrack-related transmission. This may have been because the barrack was subdivided into smaller units composed of 5 to 6 soldiers, while the lecture room contained 25 soldiers with continuously produced aerosols.

All confirmed cases, contacts, and subjects of active surveillance were monitored. All contacts and subjects of active surveillance were tested as soon as they were classified as such. Contacts were isolated for 14 days from their day of last contact with any confirmed case and were monitored for suspected COVID-19 symptoms. Subjects of active surveillance were not quarantined but were monitored for suspected COVID-19 symptoms. With the help of cluster tracking, the pattern of COVID-19 and its effects on the military units were also studied [16]. Anyone, whether a contact or subject of active surveillance, who developed COVID-19 symptoms immediately underwent a COVID-19 PCR test. Before being released from their 14 days of isolation, all contacts underwent another COVID-19 PCR test to ensure that they were not infected.

## DISCUSSION

Our study described the first COVID-19 outbreak in a military unit in Korea. During the outbreak, which started from the day when index cases were identified (July 21, 2020), a total of 22 confirmed cases were identified. The primary case was one of the instructors, who showed the earliest symptom onset among the confirmed cases in this outbreak. The last case, identified during quarantine on August 3, 2020, was a soldier who attended the lecture on July 20, 2020. On August 6, 2020, 14 days from the last exposure, the monitoring finished since no more cases were identified.

Among 5 career counseling lectures, the lecture located in Unit C showed the highest attack rate, 52.0% (Table 2). The subunit outbreak that occurred in Unit C caused additional transmission in the barrack and church, and the church caused another transmission. The IRT found that of the 5 lectures, the lecture in Unit C was the longest and was held in the smallest room (Table 2). These seemed to be the principal factors explaining the high attack rate [21], but the location of the lecturer, air conditioner, windows and ventilation, and mask-wearing by individuals might also have played a role [22-25]. For example, Unit A showed no confirmed case despite having the largest number of attendees. Although the population density of Units A and C was similar (3.26 per 1 m^2^), Unit A conducted ventilation and restricted the instructor’s movements to the platform. On the contrary, during the lecture in Unit C, the instructor moved around the lecture room, with a poorly worn mask and without any ventilation, during a 150-minute lecture. The importance of distancing and ventilation has already been reported in many previous studies [23,25,26]. Therefore, it seemed that the poor ventilation and free movement of the instructor, which led to a closer distance to the soldiers, substantially contributed to the outbreak.

The incubation period that appeared in this outbreak, 6.3 days, is similar to the known incubation period of COVID-19 [27-29]. One previous study suggested that younger cases have a shorter incubation period [30]. However, in our study, 1 of the cases showed an exceptionally long incubation period, 14 days, which increased the mean incubation period. The serial interval (5.8 days) was also coherent with previous studies [29,31].

According to the COVID-19 guidelines by the KDCA, epidemiological investigations include the 2 days before the onset of symptoms. In this study, transmission to contacts was found on the day of, the day before, and the day after symptom onset in the primary case. This result aligns with the findings of previous regarding the timing of peak infectiousness of COVID-19 [32-34].

It is important to allocate resources appropriately to find veiled cases in a large population where massive transmission is a concern. In this study, pooled testing was utilized so that many soldiers could be tested at once. Had testing been performed on individual samples, the timeline would have been greatly delayed, and additional transmissions may have occurred.

Recommendations state that decorum can be maintained by thorough supervision of military units, setting ground rules for the COVID-19 response, counseling, and disciplinary actions. The COVID-19 pandemic has provided a way for the military to increase its engagement in some health-related activities. Steps such as minimal technical military support, military-led responses, and blended civil-military responses should be considered necessary to deal with similar situations in the future [35,36].

COVID-19 outbreaks can be managed by checking whether symptoms become present through follow-up with asymptomatic confirmed cases. The military throughout Korea—in addition to the unit in Gyeonggi Province—retrospectively promoted a series of organizational changes regarding medical regulations, social distancing, and medical leadership, which substantially helped with this situation. Guidance and counseling were provided to all military units.

Despite its originality and meaningful findings, our study has some limitations. First, since the field-training took place outdoors, it was difficult to define the at-risk population due to the lack of clear places of common use. It was stated that each group had a specific time to use common use places, including the shower room, toilet, or cafeteria, but this was not, in fact, the case. Therefore, the IRT ordered screening tests for all field-training members and monitoring for 14 days to provide for every contingency. Second, since air conditioner use was identified as a risk factor that contributed to the transmission of the disease, more objective evidence could have been provided by conducting PCR testing of environmental samples. However, when the IRT arrived at the site, disinfection had already been completed.

Despite these limitations, our study is meaningful in that it is the first study of a COVID-19 outbreak reported in a military unit in Korea. The results of our research can be used to establish preventive measures against epidemics for collective facilities, including the military, dormitories, or corrective institutions. Furthermore, through the response process, the importance of cooperation between related organizations is emphasized.

## Figures and Tables

**Table 1.** Demographics of confirmed cases in a military unit in Pocheon City, Gyeonggi Province, Korea

Cases	Male	Female	Total	Age, mean±SD	No. of asymptomatic cases
Soldiers	19	0	19	20.58±0.82	2
Instructors (family)	1	2	3	51.00±1.41	0
Total	20	2	22	24.72±10.48	2

SD, standard deviation.

**Table 2.** Epidemiologic investigation and risk assessment results of a coronavirus disease 2019 (COVID-19) outbreak in a military unit in Pocheon City, Gyeonggi Province, Korea

Unit	Date and duration of event (min)	Mask usage	No. of attendees	No. of confirmed cases	Attack rate (%)	Mean incubation period (range), d	Mean serial interval (range), d	Description of site
Career counseling lecture								
A	July 14, (D^1^-3), 90	None	138	0	0.0	-	-	450.3 m^2^, standing A/C, ventilated
B	July 15, (D^1^-2), 45	None	76	0	0.0	-	-	147.6 m^2^, standing A/C, ventilated
C	July 16, (D^1^-1), 150	Poor	25	13	52.0	6.1 (3-7)	5.1 (2-6)	81.6 m^2^, ceiling A/C, not ventilated
D	July 20, (D^1^+1, D^1^+3), 150	Poor	27	1	3.7	14.0 (14)	17.0 (17)	135 m^2^, ceiling A/C, not ventilated
E	July 21, (D^1^+2, D^1^+4), 40	Poor	57	0	0.0	-	-	252 m^2^, standing A/C, not ventilated
Religious activity	July 19, (D^2^), 40	Poor	69^3^	3	4.4	5.5 (5-6)	5.5 (5-6)	224.6 m^2^, standing A/C, not ventilated
Barrack	-	Poor	6	1	16.7	3.0 (1-4)	4.0 (4)	Direct contact with confirmed case in a barrack
Field-training	-	Poor	-	1	-	-	-	Transmission through field training sharing common-use space
Total	-	-	398	19	4.8	6.3 (3-14)	5.8 (2-17)	

A/C, air conditioner.

1 Symptom onset of primary case among lecture-related confirmed cases (the lecturer); e.g., D+1 means that the lecture conducted the day after the symptom onset of the lecturer.

2 Symptom onset of primary case among church-related confirmed cases (the soldier); e.g., D means that the soldier, the source of the church-related transmission, visited the church when the day symptom started.

3 Four cases from Unit C were excluded.
